# Using a substitute species to inform translocation of an endangered territorial mammal

**DOI:** 10.1371/journal.pone.0271083

**Published:** 2023-02-16

**Authors:** Marina Morandini, John L. Koprowski

**Affiliations:** 1 School of Natural Resources and the Environment, University of Arizona, Tucson, AZ, United States of America; 2 Haub School of Environment and Natural Resources, University of Wyoming, Laramie, WY, United States of America; Sichuan University, CHINA

## Abstract

Substitute species can inform management strategies without exposing endangered species to unacceptable risk. Furthermore, experimental approaches may help to identify the causes of translocation failures, improving the chances of success. We used a surrogate subspecies, *Tamiasciurus fremonti fremonti* to test different translocation techniques to inform on potential management actions with regards to the endangered Mt. Graham red squirrel (*Tamiasciurus fremonti grahamensis*). Individuals of both subspecies defend year-round territories in similar mixed conifer forests at elevations between 2650–2750 m, where they store cones to survive over winter. We fitted VHF radio collars to 54 animals, and we monitored their survival and movements until individuals settled on a new territory. We considered the effect of season, translocation technique (soft or hard release), and body mass on survival, distance moved after release, and time to settlement of translocated animals. Survival probability averaged 0.48 after 60 days from the translocation event and was not affected by season or translocation technique. 54% of the mortality was caused by predation. Distance moved and number of days to settlement varied with season, where winter was characterized by shorter distances (average of 364 m in winter versus 1752 m in fall) and a smaller number of days (6 in winter versus 23 in fall). The data emphasized on the potential of substitute species to provide valuable information for possible outcomes of management strategies to closely related endangered species.

## Introduction

Managing endangered species to ensure their long-term persistence is a central theme in modern conservation science [1, 2]. Endangered species are broadly characterized by small population size [3], which can lead to genetic problems such as inbreeding depression, loss of genetic variation, and accumulation of deleterious mutations [4, 5], and elevated risk of extinction due to stochastic events [6]. The intrinsic value of species, as well as the services and the economic benefits that biodiversity provide to humans, are key reasons to consider conservation actions to slow or reverse biodiversity losses [7].

Translocation—the intentional movement of animals for conservation purposes—has been used as a technique to mitigate the loss and depletion of endangered species [8–10]. However, numerous examples worldwide have shown failures for translocation programs across a wide variety of species [9, 11]. Multiple factors contribute to the outcome of translocations, and often differ among species [12]. Individual characteristics (e.g. personality, physical condition, age, sex), source populations (e.g. genotype, captive vs wild born), post-release environments (e.g. predation, habitat), seasonality, and translocation techniques (e.g. handling procedures, soft vs. hard release) can all lead to varying outcomes [10, 13, 14]. Common problems with translocations include high mortality, low breeding success, and long and linear movements away from the release site [12, 13].

Outcomes of translocations can be hard to document due to insufficient data collection or difficulties in tracking the animals after translocation [12]. An experimental approach may help identify and mitigate factors that influence the outcome of translocations, which could reduce translocations failures [12], however such an approach may not be feasible for endangered taxa that, ethically, should not be used for experimentation in the wild [15, 16]. In this context, using ecologically and genetically similar species (i.e., substitute species) to test management strategies is a valuable tool to avoid disturbance of species already facing numerous challenges [17, 18]. A substitute species is defined as “species or populations studied with the assumption that they show how populations of conservation concern might respond to environmental disturbance” [17]. For this reason, the criteria used to choose an appropriate surrogate species is critical to obtain the most reliable information to support conservation efforts [17–19].

The federally endangered Mt. Graham red squirrel (*Tamiasciurus fremonti grahamensis*, [20] is a small, territorial habitat specialist [21] endemic to the Pinaleño Mountains, AZ. This subspecies was thought to be extinct in the 1950s. Only later it was rediscovered and declared endangered in 1987 under the Endangered Species Act. This subspecies lives only at higher elevations of the Pinaleño Mountains, an isolated mountain rising from the desert. After a wildfire in 2017, the population size declined to only 35 animals [22]. Given the critical status of this population, active conservation efforts that include translocation to new areas or augmentation of the population from *ex situ* bred stock should be considered. As these actions involve translocating squirrels between areas, proper translocation techniques must be developed and evaluated. Using a surrogate species to illuminate how an endangered species might respond to conservation translocation techniques is a logical management strategy.

Fremont’s red squirrel, (*T*. *f*. *fremonti*, [20] is genetically similar to the Mt. Graham red squirrel [23]. This population is found in the White Mountains of Arizona, separated by desert and grasslands approximately 110 km from the population of the endangered subspecies *T*. *fremonti grahamensis*. This separation occurred approximately 11,000 years ago at the end of the Wisconsin glaciation [24]. Both subspecies inhabit similar forests with comparable habitat characteristics, elevations, and weather conditions [25, 26]. Both subspecies also have similar characteristics to many other red squirrels: they are diurnal mammals, active year around, they have a single reproductive season focused in late spring or early summer [27, 28]. In western North America, red squirrels are territorial and vigorously defend the centre of their territory and their larder hoard (midden) from conspecifics [27, 29, 30]. Squirrels store conifer cones when available in their midden and in pits in the ground. Middens are necessary for survival as they provide cool, moist conditions that prevent cones from drying and opening [22], thus furnishing a reliable food supply over winter [27, 31]. For this reason, acquisition of a territory after natal dispersal is critical to survival and reproduction of male and female red squirrels [32, 33].

Although assessing the outcomes of substitute species may better inform conservation decisions for other imperilled taxa, these strategies have not been thoroughly evaluated in a conservation context. There is paucity of data especially about translocation of territorial small mammals, and no data that could inform translocation of the endangered Mt graham red Squirrel. In this study, we use a substitute species to examine the potential effects of seasonality, translocation technique, and body mass on the survival, post-release dispersal distances, and time to settlement of translocated animals.

We predict that 1) in winter, animals will settle closer to the release site and spend less time before settling in a new site. Winter is characterized by limited food availability [27, 31]. In winter intraspecific competition is also lower because most of the juveniles who did not establish a new midden likely experienced mortality, decreasing population density [32]. Therefore, we expect higher site fidelity in translocated animals during winter when competition is low, and animals have more time to explore the surroundings without encountering conspecifics. In this scenario we also expect a higher survival in winter, due to the reduction of exploratory movements in an unknown landscape and consequential exposure to predators. We also speculate that 2) animals will settle closer to the release site and spend less time before settling in a new site when translocated in a soft release. Soft release allows animals to learn their new environment [34, 35], therefore, mitigating their homing response after removal of the holding pen. We also expect survival will be higher for the same reason previously mentioned. Finally, we expect that 3) animals with larger body mass will spend more time to settled, as well as traveling longer distances, but they will also experience reduction in survival. Higher body mass indicates more fat reserves, constituting energy reservoir [36, 37] during the period when animals search for a new area to settle, however longer time spent looking for a settlement place expose animals to higher risk of predation.

## Methods and study area

### Study area

We translocated squirrels using two study sites in the Apache-Sitgreaves National Forest in the White Mountains (Arizona, USA). The first study site was near Big Lake (UTM coordinate: 12S 647118.39216871 3750447.4959222), while the second site was near Hannagan Meadow (UTM coordinates: 12S 655714.23295814 3723357.028484). Both sites had similar elevation, between 2650–2750 m and mixed conifer forest type. Common species were Douglas fir (*Pseudostuga menziesii*), blue spruce (*Picea pungens*), corkbark fir (*Abies lasiocarpa*), Engelmann spruce (*Picea engelmannii*), ponderosa pine (*Pinus ponderosa*), southwestern white pine (*P*. *strobiformis*), and aspen (*Populus tremuloides*) [25]. The sites are 32 km apart.

We used Big Lake in fall and Hannagan Meadow in winter due to road accessibility (the road to Big Lake is close in winter). Although it is possible season might be confounded with location, both sites have stable red squirrel populations and the same vegetation type and structure suggesting this is unlikely.

All animals were translocated to areas inhabited by other red squirrels due to difficulty of finding areas that had no established squirrels yet met the environmental requirements for settlement. Releasing animals into an occupied area, can have different outcome than releasing animals in an empty environment. However, the recovery plan for this species includes the augmentation of the population (from captive-breed individuals) following the first year of release in the unoccupied habitat. In this situation, the response of the translocated individuals from this study will provide important information on translocation strategies.

### Trapping procedure and timing

We experimentally translocated squirrels during the fall of 2018, 2019, and 2020, and during the winter of 2018/2019 and 2019/2020. In the fall, we started translocations in August until October, and we monitored the animals until the first snow (October for 2018, November for 2019). In winter we translocated the animals from January during the first winter season, and in December during the second one, until the beginning of March. We check on the translocated animals until the end of April.

We trapped squirrels with wire-mesh box live traps (Tomahawk Live Trap, Tomahawk WI: Model # 201) baited with peanuts and peanut butter. We transferred animals into a cloth handling cone [38] and marked each with unique numbered ear tag (Monel 1005–1, National Band and Tag) and coloured ear disks (1 cm Model 1842, National Band and Tag), for individual identification. We weighed each animal with a Pesola spring balance to the nearest 5g and recorded reproductive condition. Handling time was never more than 5 min to reduce stress. Before translocating an individual, each was equipped with a radio collar (SOM 2190, Wildlife Materials International, 5-7g which is less than 5% of the individual’s body mass).

During the first fall in 2018, we translocated two animals 900 m from the point of capture, and each returned to their territory within a few hours. For all other translocations, we used a distance > 3000 m.

### Hard and soft release

We implemented the hard release technique by trapping animals and transferring each in nest boxes to the new area (34 cm H, 18cm W, 23 cm L). Nest boxes were provisioned with nesting material (hay), peanuts and peanut butter with entrance holes closed to minimize visual cues known to facilitate homing [39]. We retained squirrels at their release site in their closed nest boxes during the first night at a height of 2 m.

For soft releases, we transferred individuals inside a nest box to an enclosure (152 cm H, 90cm W, 90 cm L). We provided the enclosure with approximately 400 locally collected spruce cones and a feeder with peanuts, peanuts butter, and rodent chow as supplemental feeding. We kept the squirrels in the enclosure for 5 days. After five days, we opened the enclosure and let the animals leave when they chose to do so. The sample size for soft release (n = 12) is smaller than hard release (n = 42) because we were able to obtain the required permit and IACUC approval only in late fall 2019.

We provided food supplementation to translocated animals, however during the first fall none of the squirrels used it, therefore we did not add supplemental food during fall in the following years. In winter, we provided a feeder with an initial 500 grams of peanuts, 500 g of rodent chow and 4 tablespoons of peanuts butter. We checked the feeder every two weeks, and we refilled when necessary, until April.

### Telemetry

We used digital receivers (Communication Specialists Inc. R‐1000 receiver) and yagi 3‐element directional antennas (Wildlife Materials Inc., Murphysboro IL, USA) to track each squirrel’s movements from capture to settlement, locating all individuals a minimum of once per day during the first 5 days after translocation. Subsequently, squirrels were tracked at least once per week until settlement, death, or they were missing from the study area.

We defined animals as settled when they exhibited territorial behaviour, including the rattle vocalizations or caching of cones [32], and/or or if they remained in a 100-m radius for at least 3 consecutive days (red squirrels on the White Mountains have home ranges of about 0.05 ha–Leonard and Koprowski 2009). The first day that individuals exhibited territorial vocalization or cone caching was considered the day of settlement; when staying within the same area for at least 3 days, we considered the 3^rd^ day as the settlement day. When possible, we trapped animals after settlement to monitor the change in body mass. We defined animals as missing if their signals disappeared during the season (we checked for animals consistently through all seasons) and were never detected subsequently.

We defined 3 types of mortality: unknown predator, avian predator, unknown cause. We identified avian-caused mortalities by the presence of plucked fur, a clipped tail, gut piles, and other miscellaneous parts such as ears; raptor feces or “white wash” was also often located nearby [40]. When we found only a radio-collar, we defined mortality as predation from an unknown predator. When the signal was constantly from a tree with no additional movement, but it was not possible to reach the cavity, we defined the mortality as unknown cause. When we were able to recover the body of the animal, we sent the corpse to the Arizona Veterinary Necropsy Lab, Tucson, Arizona for a detailed post-mortem analysis.

### Statistical analysis

We used Proportional Hazard Regression [41] to estimate survival for all translocated animals, and the post-settlement survival. This approach models event rates (failures) as a log-linear function of predictor variables. In our case, we used body mass (continuous variable), season and type of release (both categorical predictors). Regression coefficients give the relative effect of each covariate on the survivor function [42].

We used a generalized linear model (GLMs, [43] to examine the effect of season (fall or winter), type of release, and body mass on two response variables: time to settlement (d) and distance to settlement (m). Time to settlement was modelled using a Poisson distribution with log‐link and distance of settlement was modelled using a Gaussian distribution with identity link. We used a generalized mixed model (with Gaussian distribution, identity link, and individuals are treated as a random effect) to determine if season and translocation event influence the body mass of squirrels.

## Results

In total, we translocated 54 individual red squirrels (Table 1) and monitored post-translocation for 3 falls and 2 winters (2018–2020).

**Table 1:** Total number of red squirrels, *Tamiasciurus fremonti fremonti* translocated in the White Mountains (Arizona, USA) from 2018 to 2020, by sex, season (fall and winter), and translocation type (soft and hard).

SEASON	HARD	SOFT	TOTAL
**FALL**	21 (11 males, 10 females)	6 (3 males, 3 females)	27
**WINTER**	21 (9 males, 12 females)	6 (3 males, 3 females)	27
**TOTAL**	42	12	**54**

### Fate of translocated animals

**Fig 1:**
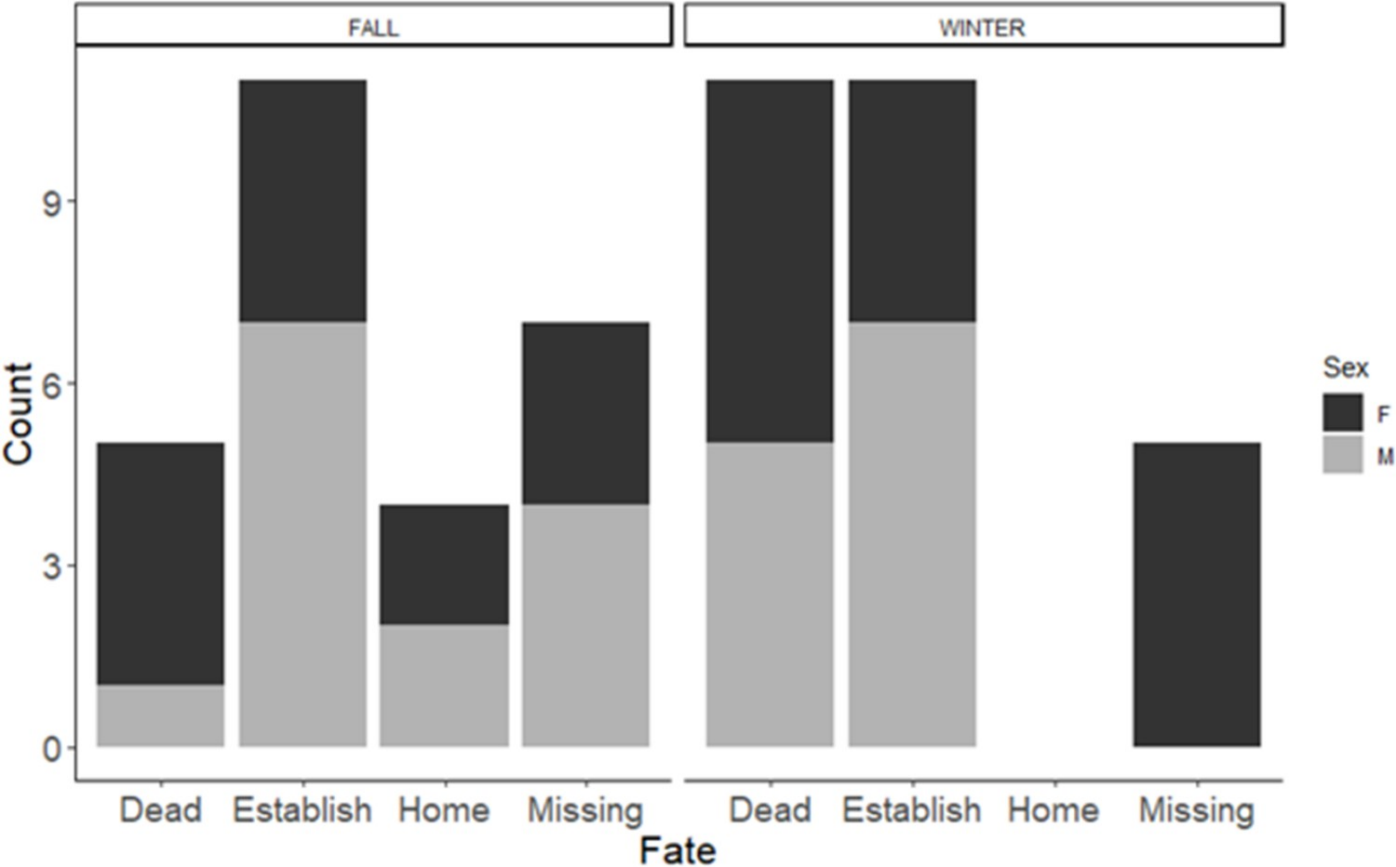
Number of red squirrels *Tamiasciurus fremonti fremonti* per different fate by sex and season (winter and fall), after translocation. The different fate has been identified as dead, establish (settled in a new area, different from the original home range), missing (when the animals was not trackable by telemetry and not possible to locate again), home (animals able to homing after being translocated).

For the 54 animals translocated, 12 (22.2%) disappeared, 22 (40.7%) settled, 4 (7.4%) returned to their original territory and 16 (29.7%) died. If we do not consider the disappeared animals for which final fate was unknown, of the 42 animals translocated, 52% settled in a new territory, 10% returned to the original territory, and 38% died prior to settling in a new territory. After settlement, 6 of 22 animals were predated, 4 in winter and 2 in fall (between 17 and 32 days after settlement). The fate of translocated squirrels was similar for both sexes and both seasons (Fig 1).

### Mortality

In total, we confirmed 22 mortalities (40%), 15 in winter and 7 in fall. 12 (54%) died due to predation (6 by raptor and 6 by unknown predator) and 10 (46%) from unknown causes. Among the unknown causes, we had one animal with the signal in a burned area, with the collar found under the snow (but impossible to recover), and 5 animals with constant signal coming from a nest, suggesting that the animals died in the nest. In fact, we were able to recover two intact squirrels from the nest where they died, one from a cavity and one from a drey. We sent, these two animals plus a squirrel found dead at the base of a tree (with intact body and no apparent signs of predation), and one found with signs of predation to a veterinary hospital for a post-mortem examination. For all these animals, the pathologist considered fat stores as adequate for this species, excluding starvation as the cause of death. Except for the animal with signs of predation, which had an ear and few fingers of the front paws missing, no signs of organ damage, or wounds were recorded.

### Survival and change in body mass after translocation

**Fig 2:**
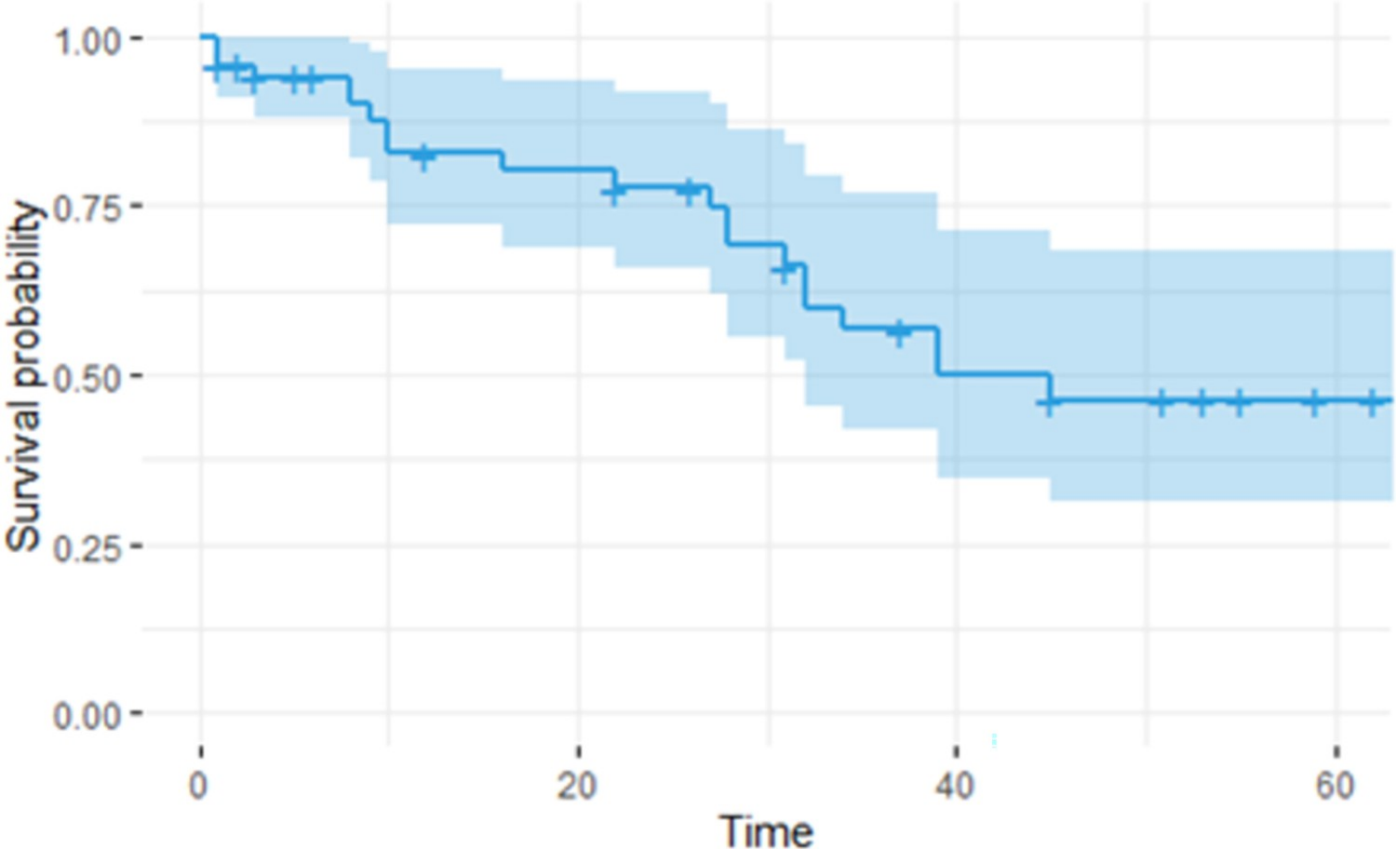
Survival curve of red squirrels *Tamiasciurus fremonti fremonti* after translocation, from the first day of release until 60 days after the release (usually corresponding also to the end of the field season). On the x-axis is represented the time in days after translocation, while on the y-axis is represented the survival probability, starting as 1 the day of the animal release and decreasing until just below 0.5 after 40 days.

Survival probability decreased steadily after translocation and stabilized below 0.5, 40 days after translocation (Fig 2). Survival did not differ between season or translocation type; however, we observed an effect of body mass on the hazard risk, where an increase of body mass corresponded also to an increase of survival (regression coefficient -0.02, Hazard ratio 0.97, CI 0.95–0.99, p = 0.039). There is an 26.3% increase in the expected hazard (probability of death) relative to one gram increased in body mass, holding season and translocation type constant.

We were able to document body mass for 6 of 11 (54%) animals that settled in winter and 4 of 11(36%) in fall. Translocation resulted in a decrease in animals’ body weight of 5.6% than non-translocated animals (t = -2.4; P = 0.02), while season had no effect (t = 1.04, p = 0.3).

**Fig 3:**
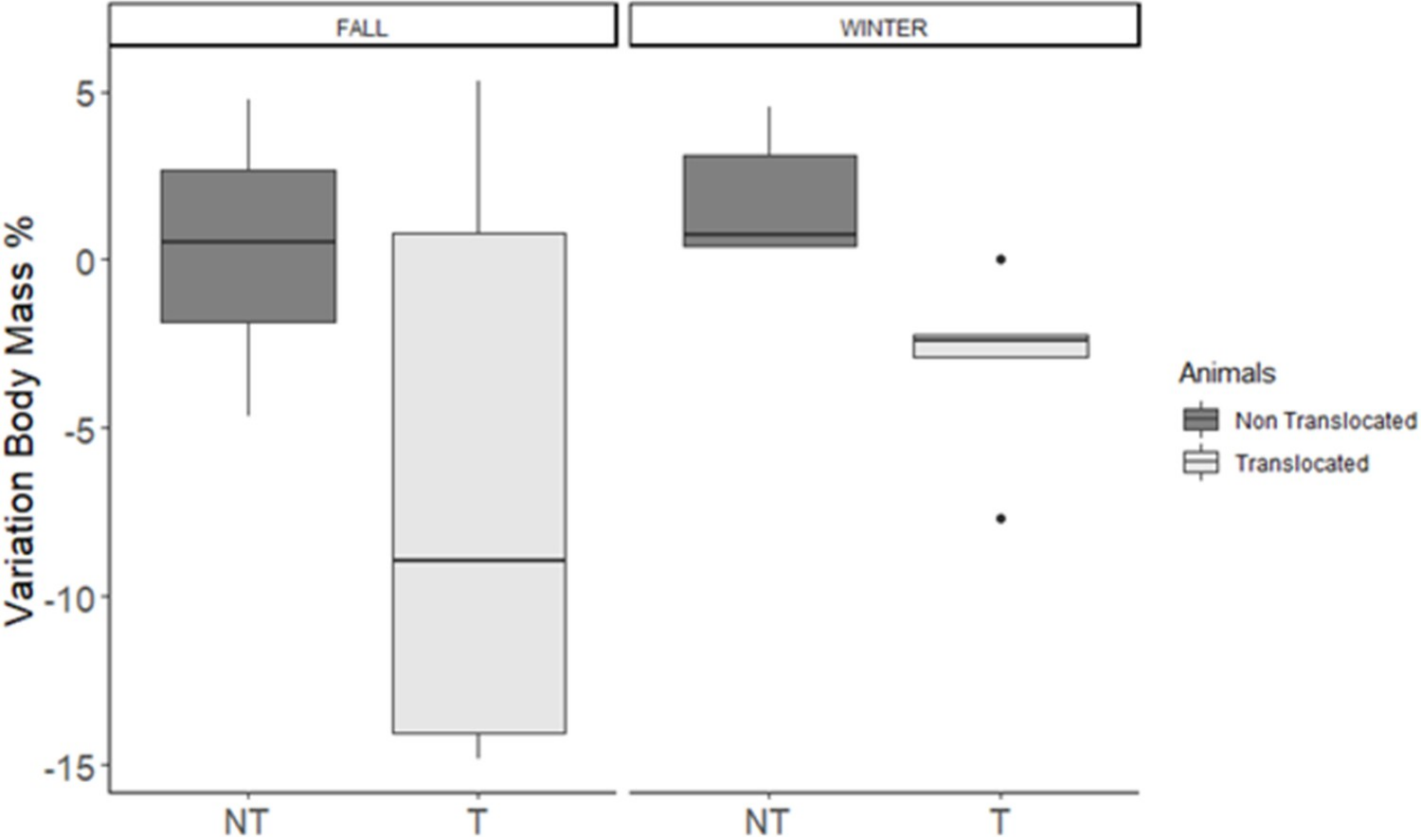
The variation in body mass of translocated red squirrels *Tamiasciurus fremonti fremonti* (N = 10), expressed as percentage of body mass change after translocation in respect to the weight at release. In dark color the variation in body mass of non-translocated animals (N = 9), expressed as percentage of body mass change between two trapping sections in respect to the weight of the first trapping event (as a control group).

On average, we observed a loss of body mass following translocation both in winter and in fall (Fig 3). We were able to document a loss of 9.5% of body weight in an individual during only 5 days of wandering behavior (released at 280 grams and 5 days later recaptured with a weight of 255 g). During this study, we documented two females with young after translocation, with the possibility that others produced offspring, but we were not able to capture to verify reproduction.

### Days before settlement and distance to settlement

**Fig 4:**
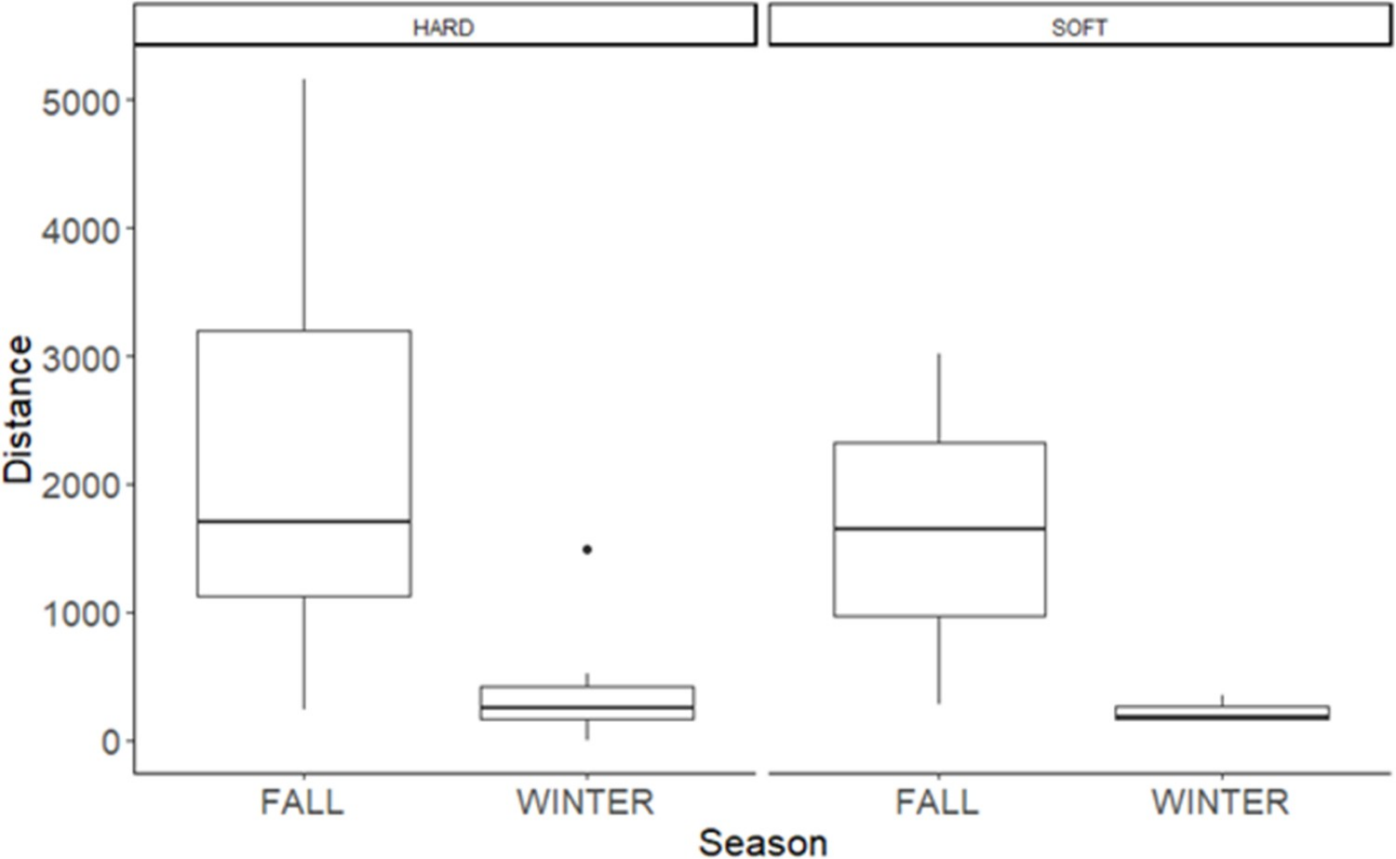
Plot of mean, sd, minimum and maximum distance of settlement site to release site (fall: mean 1752, sd 1472, maximum 5162, minimum 233). Winter: mean 364, sd 421, maximum 1494, minimum 0 (one squirrel stayed at the release site), and number of days passed before squirrel (*Tamiasciurus fremonti fremonti*) settled (Fall: mean 23, sd 13.6, maximum 57, minimum 8. Winter: mean 6, sd 2.45, maximum 11, minimum 3).

The number of days before settlement and the settlement distance were lower in winter than in fall (Fig 4, Table 2). Body mass and type of release did not affect the number of days before settlement or settlement distance (Table 2). Intensive radio-tracking during the first week post release showed that in 20 of 48 documented cases (41%), the translocated squirrel was chased away from the release site by a resident local animal. However, in winter only 7/25 (28%) were chased away, while in fall 13/24 (54%).

**Table 2:** Estimate regression parameters, standard errors, z values and P values for the generalized linear to examine the effect of season (fall or winter), type of release, and body mass on two response variables: Time to settlement (d) and distance to settlement (m) from release site.

DAYS BEFORE SETTLEMENT				
	**Estimate**	**Std. error**	**Z value**	**P value**
Intercept	2.841	0.833	3.41	0.0006
Season-Winter	-1.326	0.141	-9.34	2 e-16
Type-Soft	-0.111	0.15	-0.73	0.46
Body Mass	0.001	0.003	0.383	0.70
**DISTANCE TO SETTLEMENT SITE**				
	**Estimate**	**Std. error**	**t value**	**P value**
Intercept	4153.839	3605.345	1.152	0.266
Season-Winter	-1.849.538	527.407	-3.507	0.002
Type-Soft	-446.634	602.894	-0.741	0.469
Body Mass	-7.143	13.689	-0.522	0.608

## Discussion

We considered the effect of season, translocation technique, and body mass on survival, distance moved, and time to settlement of translocated animals. As we predicted, during winter animals showed higher site fidelity than in fall. Moreover, the distance and number of days before settlement were reduced in winter, likely due to limited food availability and reduction of intraspecific competition. Translocation techniques (soft vs. hard) did not influence days and distance to settlement site. Contrary to our prediction, season and type of release did not affect animal survival. However, survival was higher for animals with higher biomass.

### Survival after translocation: the cost of the wandering phase in a “forced dispersal”

Translocation forces animals to effectively experience dispersal. During dispersal, we observe three phases: a) Initiation, when an individual leaves its home area; b) Wandering, when the dispersing individual searches for new areas before settling; and c) Settlement, when the individual settles in an area [44]. Likewise, translocated animals leave the release site and wander in unfamiliar areas until they select a new place to establish. In those terms, translocation can be view as a “forced dispersal” [45]. The lack of familiarity with the environment likely causes a decrease in survival, frequently associated with an increase of predation risk, during dispersal [32].

In this study survival decreases constantly with time and stabilized after about 40 days from the translocation event, when most of the squirrels who survived, usually settled/build a new midden. Middens are a fundamental component of red squirrels’ territory; they provide shelter, escape from predators and a reliable food supply during winter [46]. In fall, we observed squirrels using old middens, or creating a new midden by initiating caching of cones in a new area. During winter, some individuals settled in an area, using artificial food, stealing cones from other middens, and moved into an existing midden only later in the season. For this reason, we can consider this event the completion of the post-translocation period. This is important information to have when planning the duration and the effort of post monitoring program. They have been long recognized as crucial for any component of translocation [12], however are also difficult to sustain for a long period of time due to lack of funding and/or animal behavior [47]. Monitor efforts following translocation in red squirrel seems necessary especially in the first 40 days after release, or at least until animals settled in a new midden, with allowing a decrease in the intensity due to animals being less likely to move.

The mortality rate of animals during the wandering phase (between 0 and 57 days) in this study was 42% (excluding the missing animals). This value is not too different from the translocated Eurasian red squirrels (*Sciurus vulgaris*), which in Belgium had 50% (females) to 67% (males) mortality rates in the first months following release [48]. If we consider translocation as a “forced dispersal”, we could expect similar mortality values across dispersers. However in the literature we can found a wide range of mortality rates during natal dispersal in America red squirrels (lasting not more than 2 months): 22% mortality in the forests of the Athabasca Sand Hills region of Alberta—Canada [32]; 27% in the Yukon—[49]; 59–67% in Rochester, Alberta—[33, 50]; and 23% for the subspecies, Mt Graham red squirrel—[51]. Furthermore, natal dispersal is always associated with juveniles, while the forced dispersal of the individuals translocated is associated to adult individual. The age of the individuals translocated could also affect the success of the translocation [13]. We highlight the need of further research to investigate how the translocation of juveniles can conduct different outcomes.

Season did not affect the overall survival of animals, however the frequency of different causes of mortality seems to change across season. The main cause of mortality was predation, which represents a widely documented cost associated with dispersal among mammal species [52]. In our study, predation accounted for at least 54% of deaths, mainly due to avian predators. Raptors are also the major cause of deaths for juveniles and adults in Mt Graham red squirrels in the Pinaleño Mountains for settled animals [40] as well as during dispersal [51] (they cause 75% of yearly deaths in juveniles and 65% in adults). Although the most common cause of mortality was predation, 10 translocated animals died from unknown causes, mostly in winter. In some situation the mortality occurred in a nest. A tentative explanation for at least some of these unknown cases of mortality is stress, an inevitable component of translocation [53]. Translocation alters stress physiology and chronic stress is potentially a major factor in translocation failure [54]. However, a more thorough necropsy would be necessary to determine the exact cause of death and elucidate the possible actions taken to reduce mortality. The only alternative to decrease mortality in this species would be to reduce risk of predation, perhaps limiting the number of days spent before settling in a new habitat.

### Seasonal effects on dispersal: food availability as limiting factor in winter

Seasonality in environmental factors can represent an important factor for the success of translocations. Here we report an influence of season on the number of days spent by translocated individuals to settle as well as on the distance between release and settlement site. In winter, animals settled faster and closer to the release site than in fall. The limited movements of animals during winter, can also explain the absence of homing behavior in winter. Two factors can explain the different pattern of movement in the two seasons: Food availability and distribution, as well as pressure from intraspecific competition.

Food distribution and availability can limit the movement of the animals. In fact, the cones cached by the red squirrels in middens during the fall are fundamental to sustain the animal during winter. The only reliable food source accessible to translocated animals in the coldest season is the artificial food supply provided at the release site [27, 31]. Therefore, this situation can reduce the wandering behavior of animal, which can in turn decrease risk of predation. The habitat available for Mt Graham red squirrel, in the Pinaleño Mountains after the fire, is highly fragmented and only some patches have good connectivity [22]. Therefore, long distances travelled by translocated animals, can cause dispersal into areas with no habitat, further increasing the mortality risk. In this contest, translocation during winter, can provide a limiting factor to the dispersal distance.

Differences in the intraspecific competition between seasons, can also explain the difference on site fidelity of squirrels after translocation. The suitability of the settlement site is also determine by the density of conspecifics within that habitat [55]. In territorial red squirrels, intraspecific competition in fall is high because juveniles disperse from their natal area and try to establish a new midden, or to take a resident’s territory [56–59]. Since the behaviors associated with dispersal and competition to obtain a territory are costly, juvenile mortality between fall and the onset of winter will be high and population density will decrease [32]. Proceeding with translocation of animals in winter, can therefore allow animals to explore the surroundings with lower chance to encounter territorial conspecifics. In fact, during our study, we observed a higher number of aggressive interactions during fall than winter, with local squirrels entering the nest box of translocated animals to chase from the translocation site.

Planning the translocation of the endangered species, needs to address the distribution of resident conspecifics, due to their key role in the translocation outcome [60]. The results here obtained are not a good prediction on the actual behavior of translocated animals if the endangered species will be reintroduced in empty areas. In fact, we were not able to find a place characterized by good squirrel habitat and without any individuals. Without conspecific, we might observe higher retention also in fall, and/or shorter movements before settlement. However, in the recovery plan for Mt Graham red squirrel, translocation is also proposed as a method for the augmentation of the population following the first year. In this context, the information obtained by our study, can provide insight to the potential outcomes of the translocation. Considering the importance of the pressure exerted by conspecific, further research is needed to determine how different local densities affects retentions and how multiple translocation events in the same site can negatively impact those individuals that have already settled (because of a sudden increase of abundance of the local population). Red squirrels during natal dispersal often choose territory sites close to familiar neighbours [57, 59] and their presence reduces rates of territorial rattle calls and increases time spent in the nest [61]. Therefore, further research could determine if a solitary and territorial species such as the Mt Graham red squirrel, might benefit translocation in groups of squirrels, to keep stable neighbours [62].

### No difference between hard and soft release

We used both soft and hard releases in the translocation of red squirrels. Soft release provides translocated animals the opportunity to acclimate to their release site [34, 63, 64]. In our study, the type of translocation (hard/soft) did not affect survival, distance to settlement site, or number of days to settlement in red squirrels. The smaller sample of soft release animals relative to hard release animals, might have affected our results. However, multiple cases exist where no difference in outcomes have been documented between hard and soft releases, as for the case of brushtail possums [65], hare-wallaby [66], and translocated colonies of black-eared miner [67].

The absence of benefit of soft release on site fidelity, can be attributed to the pressure exerted by resident conspecifics. In fact, most translocated animals (41%) were chased away by residents in the first 3 days after release. During the fall, the pressure exerted by the red squirrels present in the release area was intensified, where resident animals were exploring the enclosure and were making territorial calls from the top of the enclosure directly towards the translocated animals within the enclosure. This solitary territorial species benefits from having stable neighbors [61], at a point where animals avoid settlement in new empty middens even when they occupy poorer quality territories [68]. For this reason, translocating single animals can cause the disruption of social relationship with territorial neighbours, which could have an impact on survival and behavior of translocated animals [62], as we discussed in the previous section.

Our results can provide the potential outcomes if translocation will be used for future augmentation efforts on Mt Graham red squirrel, but not necessarily during the first reintroduction, as we have already mentioned. From this study, soft or hard release did not provide any specific benefits, leaving the choice to this method to reflects other needs (for example time or budget). However, if in the future will be tested translocation of group of individuals, proceeding with soft release will allow animals to have time to recognize their neighbours and get use toe the new place.

### Effects of body mass on translocation outcomes: the advantages of higher body mass

Body mass affects survival, with heavier animals being more likely to survive, though it does not affect distance moved or time to settlement. Translocation might cause increased physiological stress in animals, which in turn can reduce their body mass [54]; and can also increase energetically costly behaviors, such as wandering [44]. In fact, we observed a loss of body mass following translocation both in winter and in fall. After translocation, animals find themselves in a totally new environment, where they need to learn where to find food and shelter, and what are the areas with higher risk of predation. Finding good quality nest sites can also influence energy consumption for thermoregulation, particularly when weather conditions are extreme. For this reason, when planning to translocate the endangered species, it is better to select for animals with higher body mass, because they are better equipped to deal with low food intake during the wandering phase after translocation.

### Lessons from using substitute species

Our results emphasize the potential of substitute species to provide valuable information on possible outcomes of management strategies for closely related endangered species. In our case, the estimated population size of the endangered species when we planned this study (fall 2017) was only 35 individuals [22], making it impossible to test any management strategies directly on the remaining animals without multiple risks associated with any form of manipulation. Despite the potential offered by substitute species, not many studies used a non-endangered relative species as a substitute for an actual target species [69]. The outcomes of this study are meant to assist the planning of management strategies to restore the population of Mt Graham red squirrel, elucidating potential outcomes of different translocation strategies as well as underling potential factors and or problems that could represent a key in the retention and survival of the animals to the release site. We encourage the use of substitute species for developing and improving management strategies, including translocation, to achieve an increase in the likelihood of success [70], as well as to design individual marking or monitoring methods, [71], as well as aid in the identification of key problems that can arise during phases of management plans that may affect the related target species [72].
